# Supplementary material for: Are carbon emissions trading and green financial instruments synergistic? -Comprehensive quantitative research based on content analysis
Source: PLoS One. 2024 Mar 7;19(3):e0298601. doi: 10.1371/journal.pone.0298601 (PMC10919601; doi:10.1371/journal.pone.0298601)
Supplement: S1 Data — (ZIP) [file pone.0298601.s001.zip › Data/地级市Z_F工作报告.zip!/║ú─╧/╚2╤╟╩╨/└┤╫╘╣1⁄2╓┌║┼í╛┬φ┐╦╩2╛▌═°í┐_2013.docx]

各位代表：

现在，我代表市人民政府，向大会报告政府工作，请予审议，并请市政协委员和其他列席人员提出意见。

2012年工作回顾

过去的一年，国内外经济形势严峻，三亚面临着较大的发展压力。在中共三亚市委的坚强领导下，我们以迎接和学习十八大精神为动力，以科学发展观为指导，按照稳中求进的总基调，全力以赴稳增长、调结构、保民生、促稳定，取得了来之不易的成绩。全市地区生产总值330.75亿元（含农垦，下同），同比增长9.3%，其中第一、二、三产业分别增长7.1％、9.0％和9.8％。全市地方公共财政收入60.25亿元，增长20.4%。城镇居民人均可支配收入23295元，增长13.8%。农村居民人均纯收入8825元，增长16.4%。全市物价总水平保持基本稳定。省里下达的节能减排任务预计能够顺利完成。

一年来，我们主要做了以下工作：

一、全力抓好项目建设和投资增长

围绕“项目建设年”各项要求，细化落实“六个一”责任模式，着力提高征地拆迁和行政审批工作效率，推行重点项目属地管理，加强项目督导检查，实行项目推进工作目标奖惩制度，取得积极成效。72个市重点项目完成投资300.7亿元，全市固定资产投资完成430.34亿元，增长21.0%。河东、海棠湾等区镇超额完成重点项目投资任务，为全市投资增长做出了重要贡献。

二、努力保持主导产业平稳健康发展

旅游方面，我们对具有突出贡献的酒店、旅行社，对邮轮停靠三亚，对航空企业和重点旅游项目实施了政策扶持。在继续推进海棠湾等度假区及景区景点建设，增加旅游产品供给的同时，开展旅游市场专项整治，强化假日旅游管理，积极构建旅游诚信管理平台，优化了旅游环境。2012年全市接待过夜游客1102.22万人次，增长8.0%；实现旅游总收入192.22亿元，增长11.0%。房地产方面，我们鼓励开发经营性产品，并在北京等重点城市开展了旅游房地产促销。2012年全市商业用房批准预售面积增长5.6倍，在商品房批准预售面积中所占比重从6.3%上升到33.7%。在保障性住房的带动下，全市房屋销售面积180.03万平方米，增长10.0%。

三、加快推动产业结构优化调整

2012年，我们加大了对新兴产业的扶持力度，中铁子悦薹城市综合体基本建成，南航三亚总部基地综合楼项目加快建设，美亚旅游航空等公司总部先后入驻。阳光人寿保险总部、亚洲金融合作联盟落户三亚，国家开发银行三亚分行、民生银行三亚分行、三亚农商银行获批筹建。2012年金融业增加值达到23.64亿元，增长64.6%。免税新政的实施刺激了旅游购物，商贸产业进一步发展，全年实现社会消费品零售总额104.8亿元，增长21.8%。创意产业园、省动漫产业基地、惠普海棠湾基地等项目建设稳步推进。崖城镇保平村入选首批中国传统村落，三亚千古情城等文化产业项目加快建设，成功举办了沃尔沃环球帆船赛三亚站比赛、三亚国际沙滩音乐节、三亚国际婚庆时尚周、首届三亚国际玫瑰节等活动。加快推进崖州中心渔港、中科院三亚深海科研所建设，组织开展南沙渔场捕捞作业，推广优质水产品种养殖，海洋经济取得新发展。继续加大支农惠农力度，完善农业科技服务，紧抓冬季瓜菜和热带水果生产，积极培育花卉产业，全市农林牧渔业实现总产值70.43亿元，增长7.1%。工业生产呈逐季加速趋势，全年完成工业总产值58.03亿元，增长17.2%。

四、着力强化节能减排和生态环保工作

严格实施固定资产投资项目节能审查，加强对重点用能单位、减排单位和重点污染源的监督管理。荔枝沟水质净化厂建成投入运行，三亚市新城水质净化厂、镇村垃圾中转站等项目加快推进，城镇生活污水集中处理率达到84.2%，生活垃圾无害化处理率和生活垃圾渗滤液达标处理率保持在100%。鼓励和推广新能源应用，南山电厂500KW太阳能光热发电项目投入运行，夏日百货商场300KW屋顶太阳能光伏发电工程已完成，全市新增太阳能热水系统民用建筑应用面积210万平方米。通过了节水型社会建设试点验收。强化海洋规划编制和管理，对三亚湾等六大港湾陆源入海排污口进行了调查，设置监测站位32个。大力实施“绿化宝岛”工程，全年完成造林3.55万亩，全市森林覆盖率达68%。建立了“十二五”农村环境综合整治市级项目库。吉阳镇获评“国家级生态乡镇”。

五、继续加强城乡建设与管理

认真落实“科学规划年”各项部署，完成了17项控规、9个专项规划、各镇总规和镇区控规的编制任务，基本实现中心城区法定控规全覆盖，村庄建设规划覆盖率达到60%。凤凰机场三期扩建、西环铁路三亚段等项目稳步推进，绕城高速凤凰出口路已建成，迎宾路与绕城高速实现互通，新风桥重建、榆亚路改造、春光路丰兴隆段改造已完成，对城区部分交叉路口进行了优化改造。城乡电网建设和天然气管道等项目进展顺利，水居巷、榕根村等城中村改造稳步实施。努力提高城市管理水平，推进市容和环境卫生等管理权下移，开展全民参与城市管理活动，数字化城市监控系统全面投入使用，数字化城市管理系统投入示范运行。继续开展“铁锤行动”，拆除违章建筑约70万平方米。农村公路与桥梁改建维修工程有序推进，病险水库除险加固、小型农田水利项目年度建设任务顺利完成，大隆灌区西干渠工程进展较快。6个海棠湾风情小镇在去年开工建设，新农村综合改造建设试点加快推进。建成育才镇农村饮水安全工程，解决了5000多人的饮水安全问题。

六、进一步深化改革开放

积极开展服务业综合改革试点工作，大力支持服务业发展。截至去年底，我市服务业信贷余额215.37亿元，同比增长18.2%，占同期各项贷款余额的70.6%。对市属国有企业进行调整重组，新组建了海南航程旅游发展股份有限公司。深化农村综合配套改革，出台了《三亚市集体建设用地开发利用管理暂行办法》。强化涉农金融服务，2012年发放涉农贷款18.7亿元，农业保险试点品种增加到4个。积极推进政府财政预算管理制度改革，认真开展预算听证和区镇国库集中支付扩面工作。深化强镇（区）扩权改革，各区镇在项目管理、城市管理、社会管理等方面的主体作用得到加强。医药卫生体制等方面的改革取得新进展。充分利用第八届泛珠大会等平台，积极组织招商活动。2012年我市实际利用外资2.36亿美元，完成进出口总额突破1亿美元。不断完善外事管理与服务，深入开展国际交流，提升了城市国际知名度。

七、全面推进民生和社会事业发展

2012年，我们安排了49.05亿元用于十大重点民生项目。住房保障方面，共建设各类保障性住房9554套，完成农村危房改造1758户。就业方面，新增城镇就业岗位33876个，转移输出农村劳动力15341人，动态消除零就业家庭，城镇登记失业率为1.91％。社保方面，全市新型农村养老保险参保率达到98.8%，城镇居民养老保险参保率95.8%，城乡居民医疗保险参保率99%，各项保障水平均有不同程度的提高。物价方面，大力实施“菜篮子”工程，新建了一批农副产品平价商店和平价专营区，积极开展农超对接，加强价格监测预警和监督检查，发放物价上涨联动补贴3.48亿元。教育方面，扶持优质民办幼儿园发展，推进中小学校布局调整，扩大普通高中办学规模，支持高等院校加快发展。学前三年教育毛入园率达到74.8％，高校在校生达到4.1万人。医疗卫生方面，基层医疗卫生服务体系进一步健全，解放军总医院海南分院正式开诊，市中医院获评“三级甲等中医医院”。社会管理和服务方面，各区镇便民服务中心已挂牌运作，在18个社区实行了“网格负责制”新型管理模式。严厉打击“两抢一盗”等犯罪活动，加强流动人口及出租屋管理，完善社会治安防控体系，初步建立起视频监控巡逻网。强化食品药品安全监管，开展重点行业和领域安全生产专项整治，“平安三亚”建设取得新成效。倡导优生优育，完成了省里下达的人口计生工作目标任务。认真处理群众来信来访，深入开展大接访、大下访等活动，畅通信访渠道，各种矛盾纠纷得到及时排查调处。深入推进双拥共建工作，再度荣获“全国双拥模范城”称号。民族、宗教、侨务、统计、档案、口岸、地震、人防等工作取得新成绩，妇女儿童、青少年、老龄、残疾人等事业取得新进步。

八、不断加强政府自身建设

自觉接受人大、政协的监督，一年来办复市人大代表建议、批评和意见135件、市政协委员提案135件，办复率100%，满意和基本满意率达到94.8%。加强依法行政，行政复议案件量比上年减少26%。对行政审批项目进行清理和规范，2012年市政府政务服务中心共办理各类审批及服务项目16.3万件，平均办理时间从上年的4.9个工作日缩短为3.2个工作日，当场办结率达到50.3%，按时办结率100%。狠抓工作督查督办，认真开展绩效考评，实行财政预算项目绩效监督分析，严格执行各项问责制度，提高了行政效能。加强政府信息公开、政府采购管理和投资评审管理，规范公共资源交易管理，围绕工程建设等重点领域开展专项治理，严肃查处各类违法违纪案件，解决了一批损害群众利益的突出问题。

各位代表，过去的一年，面对严峻的经济形势，我们努力克服各种困难，扎实推进各项工作，取得了来之不易的成绩。这是省委、省政府和市委正确领导的结果，是全市人民共同奋斗的结果。在此，我谨代表市政府，向全市各族人民，向各民主党派、各人民团体和社会各界人士，向驻三亚解放军、武警官兵、公安司法干警，向中央和省驻三亚各单位，向所有参与、支持、关心三亚建设发展的国内外朋友，表示衷心的感谢！

回顾2012年，我们也清醒地看到，三亚经济社会发展中仍然存在一些问题，政府工作上还存在不足。一是全市地区生产总值、旅游接待过夜游客等指标未能完成预期目标。这虽然与国内外经济环境有很大关系，但也暴露出我市产业结构上的问题。传统优势产业的结构升级和新兴高增值产业的培育壮大，仍然是我们当前面临的重要任务。二是征地拆迁难、土地供需矛盾突出、行政审批效率不高、前期工作不充分等影响项目建设的难点问题尚未得到有效解决。三是城市功能亟待完善，公共服务供给不足，城市管理的常态化机制仍需健全，城市的舒适度不够高。四是社会事业特别是教育、文化事业发展总体水平偏低，这也在一定程度上制约了高增值产业的发展。五是政府层级管理有待进一步完善，各区镇的发展能力需要进一步提升，个别部门和人员服务企业、服务群众的意识还不够强。对于这些问题，我们将在今年的工作中努力改进和解决。

2013年主要任务

2013年是全面贯彻落实党的十八大精神的开局之年，我市既面临着加快发展的重大机遇，也面临着复杂多变的发展环境。政府工作的总体思路是：深入贯彻落实十八大精神，紧紧围绕国际旅游岛建设，深化改革开放，加快转变经济发展方式，大力推进生态文明建设，稳步实施文化强市战略，不断改善人民群众生活，努力建设更加卓越、更加和谐的三亚。

今年全市经济社会发展的主要预期目标是：地区生产总值增长10%，全市固定资产投资增长20%，地方公共财政收入增长15%，旅游接待过夜游客增长8%，社会消费品零售总额增长20%，城镇居民人均可支配收入增长10%，农村居民人均纯收入增长11%，城镇登记失业率控制在3%以内，居民消费价格涨幅控制在5%以内，完成省里下达的节能减排任务。

为实现上述目标，要重点抓好以下几个方面的工作：

一、加快转变经济发展方式

在增强产业实力和扩大内需的同时，积极优化产业结构和需求结构，推进体制创新，统筹城乡发展，实现优质高效增长。

（一）继续调整产业结构。一方面，要巩固提升旅游、房地产等主导产业发展水平。加快海棠湾、亚龙湾、南山等旅游片区的建设。特别是海棠湾，今年要完成基础设施重点项目投资15亿元和现代服务业重点项目投资50.5亿元。大力开发邮轮、游艇、海上娱乐、水上观光、会展、婚庆等特色旅游产品。积极推进旅游标准化城市建设，在南山等重点区域设立7个旅游咨询服务站，进一步完善12301旅游资讯公益服务平台的运行。继续探索完善旅游城市出租车管理模式。有针对性地开展旅游市场环境综合整治，规范市场经营秩序。鼓励发展经营性房地产，适度开发普通商品住宅。着力加强房地产项目配套设施建设。规范房地产交易管理，简化交易手续，促进房地产业平稳健康发展。另一方面，要加快打造新的增长点，培育壮大总部经济、金融产业、智慧产业、海洋产业等新兴产业。积极推进阳光金融广场、南航三亚总部基地综合楼等项目，引导大企业进驻中铁子悦薹商务写字楼，逐步形成集聚效应。完成国家开发银行三亚分行、民生银行三亚分行和三亚农商银行的设立工作，大力支持其业务发展。加快创意产业园、高新技术产业园等园区基础设施建设，引进知名企业和重大项目。开工建设省动漫产业基地三亚动漫城，中兴、用友、惠普云计算与3D动漫产业园等项目要取得重要进展。加快崖州中心渔港建设，更新改造20艘南沙生产渔船，积极开发外海渔场，建立赴南沙捕捞常态化服务保障机制。继续推进海洋牧场建设，合理发展深水网箱养殖。积极推动南海油气资源开发服务基地和南海旅游服务基地建设，高效利用岸线资源，在不破坏生态环境的前提下，稳妥实施填海项目。

（二）促进投资和消费稳定增长。一方面，要继续抓投资、上项目，扩大投资规模。今年安排市重点项目97个，年度计划投资316亿元。海棠湾威斯汀度假酒店、海棠湾洲际度假酒店等一批高星级酒店项目要在年内竣工；凤凰机场三期扩建、西环铁路三亚段建设、鹿回头半岛开发、美丽之冠大酒店、三亚湾红树林度假会展酒店、太阳湾高级度假区、半岭温泉旅游度假区以及水居巷、榕根村旧城改造等项目要加快推进；西部供水二期工程、崖州湾综合开发区、红塘湾旅游度假区等项目要开工建设；凤凰岛国际邮轮港码头二期和临春城中村改造等项目要抓紧完成前期工作。继续抓好项目储备，优化储备结构，并积极争取地债资金支持。进一步发挥区镇的管理作用，努力破解征地拆迁等难点问题，妥善安置群众，切实保障其合法权益，坚决打击抢种抢建、违法阻工等行为。落实“六个一”责任模式，提升行政审批效率，强化项目服务和监督检查。另一方面，要积极引进和培育消费。创新方式，继续开展旅游和房地产宣传促销，提升推介效果。大力发展商贸产业，用好用足购物免退税政策，进一步加强市内免税店建设和招商，加快推进海棠湾国际购物中心、三亚沃天堂地下商业综合体等项目。积极培育文化、体育、保健、信息等消费热点，规范家政服务行业市场管理。

（三）深入推进经济体制改革和对外开放。继续实施市属国有企业调整重组，通过组建集团公司等方式，整合优质资源，培育龙头企业。积极推进国有企业股份制改造，支持大小洞天公司等企业上市。优先引入业绩优秀、信誉良好的民间投资主体，发展混合所有制经济。支持和引导非公有制经济发展，继续实施中小企业成长工程，完善中小企业信用担保服务。进一步规范行业协会、中介组织发展。继续开展服务业综合改革试点，积极开发“好运三亚”旅游娱乐竞猜游戏等项目。进一步深化财政预算编制改革，试行编制绩效预算，扩大预算听证的范围和内容。继续实施“开放带动”战略，积极融入泛珠三角等区域合作，充分利用各种平台，加强招商引资，扩大贸易进出口。

（四）着力统筹城乡发展。进一步完善城乡规划体系，推动城乡发展一体化。在基础设施上，加快公交场站、城市停车场以及春光路海螺段等道路工程建设，争取尽早实施三亚大桥重建、月明桥建设等项目。推进城乡电网建设和城镇供水管网扩建，着力完善教育、医疗、文体、商业、金融等服务设施。积极推进育才省级特色风情小镇示范镇和海棠湾风情小镇建设，加快新农村综合改造建设试点，加强村镇规划建设管理。继续完善绕城高速公路连接线和出口路建设，完成农村公路27项通畅工程和农村桥梁改建维修各项任务，全市行政村公交覆盖率要达到80%。开工建设大隆灌区东干渠工程，大力实施病险水库除险加固、田洋灌溉及排涝渠道改造等水利项目。积极开展农村饮水安全、改水改厕、沼气建设等工作。在产业经济上，加大农业投入，积极发展热带水果、热带花卉、冬季瓜菜、南繁育制种等特色产业。推进国家冬季瓜菜基地、亚龙湾兰德玫瑰风情产业园、吉阳镇热带兰花生产示范基地等项目建设。完善农民培训体系，扶持农民专业合作组织发展。引导企业和农民专业合作组织建立标准化基地和现代化加工配送体系，开工建设崖城农产品加工出口基地二期项目。加强农产品质量安全检验检测。在体制机制上，进一步深化农村综合配套改革，促进土地、人力、资金等资源在城乡之间合理配置。全面完成农村宅基地登记发证，加快农村集体土地共有宗地分割确权登记发证。打击农村土地非法买卖，加强土地流转指导、协调、服务和管理，强化集体经济组织、新型村民自治组织的作用，发展农民股份合作。继续推进农村土地产权制度改革试点和城乡建设用地增减挂钩试点，全面推进林改综合配套改革。完善失地农民社保体系和就业培训体系，为推进农业转移人口市民化创造条件。建立农村征信系统，继续强化小额信贷、农户联保贷款和农业保险等金融服务。在城乡管理上，继续下移管理权，落实网格化管理机制，加强城管日常巡查执法。完善数字化城市管理系统、监控系统的运行，扩大全民参与城市管理活动的效果。着力优化道路交通组织管理，改善城市交通环境。坚决打击违法建设行为，完善拆除违建流程，加强新增违建监控。认真实施三亚市国家卫生城市长效管理机制，积极创建卫生镇、村。

二、大力推进生态文明建设

以实现永续发展为目标，坚持把建设生态文明、保护生态环境放在经济社会发展的突出位置。

（一）规范土地和海域资源开发管理。严格落实耕地保护制度，完成基本农田划定工作，积极实施土地整理项目、耕地占补平衡项目，加强土地资源统筹管理。强化土地开发监管，严防非法占地、闲置土地等行为。修编《三亚市海洋功能区划》，强化海域资源管理，推行海域使用权公开招标拍卖，促进海域资源高效配置与合理利用。

（二）加大节能减排工作力度。进一步完善节能目标责任制和节能考核评价制，加强固定资产投资节能评估和审查，全面开展重点用能单位能源审计，抓好工业、住宿业、交通运输、公共机构等重点领域节能。严格执行新建建筑节能50%的强制性标准，推广太阳能建筑应用。开工建设三亚生活垃圾焚烧厂，积极推进城市污水配套管网及三亚湾、三亚河区域城市雨污水分流改造工程，在年内建成三亚市新城水质净化厂，试运行三亚餐厨垃圾处理项目。强化重点污染源巡查监管，推进在线监控设施建设。继续开展垃圾分类收集和再生资源回收利用体系建设试点。加大机动车尾气污染监管力度，对排放超标的机动车实行区域限行。防治畜禽养殖污染，加强农业面源污染治理。

（三）强化生态工程建设和环境保护。推进“绿化宝岛”工程，确保完成海防林建设、城市森林建设、村庄绿化、通道绿化、河流水库绿化以及生态经济兼用林等工程造林2.67万亩。继续开展森林生态效益财政补贴试点工作。加强城市公园建设与管理，积极推进三亚河红树林保护区定界埋桩。切实加强饮用水水源地环境监控和保护。大力开展海洋环保宣传，认真实施海监巡查特别是对重点海域和海洋工程建设项目的巡查。继续完善防汛抗旱指挥系统，尽快建成红树林地震避难场所。积极开展应急行动演练，增强防灾减灾和应急救援能力。

三、稳步实施文化强市战略

挖掘城市文化内涵，加强精神文明建设，推动文化产业和文化事业发展。

（一）着力培育文化产业。深入推进文化体制改革，鼓励和引导社会资本兴办文化企业，开发文化项目。创造良好的产业发展环境，吸引外来人才，大力开展文化创新活动。完成三亚千古情城的建设，继续引进有实力、有特色的文化产业项目。办好UIM近海一级动力艇世界锦标赛中国三亚大奖赛等活动，继续引进品牌文体活动与赛事。积极开发三亚独特的生态文化、海洋文化和少数民族文化，加强非物质文化遗产的挖掘、保护和利用。

（二）加快发展公共文化体育事业。开工建设市群众艺术馆，充分发挥市体育中心的功能，加强城市公园和社区体育设施建设。实施文化惠民工程，加快完善社区文化中心和乡镇文化站设备配套，继续推进农村文化室、农家书屋、篮球场、休闲广场和多功能服务中心等设施建设，完成广播电视“户户通”工程建设任务。广泛开展群众性文化活动、全民健身运动，认真做好文化下乡工作，丰富群众文化体育生活。

（三）积极推进文明城市建设。深入开展社会主义核心价值体系学习教育，加强城乡居民道德素质建设，倡导爱国、敬业、诚信、友善，倡导科学、健康、文明的生活方式。加强法制宣传教育，提高城乡居民法律素质。积极开展三亚“文明大行动”，开展旅游文明素质教育实践和“共铸诚信三亚”活动，着力构建和谐礼让的公共秩序。积极塑造开放包容的城市心态和形象，增强广大市民的主人翁意识和外来游客的归属感，促进和谐文化建设。

四、加强以保障和改善民生为重点的社会建设

认真实施重点民生项目发展规划，落实民生实事，加强和创新社会管理，让发展的成果更多地惠及人民群众。

（一）优先发展教育事业。积极推进义务教育均衡发展，完成城区中小学校标准化建设，改扩建3所农村寄宿制学校，建成省级规范化学校6所、市级规范化学校51所。加快各区镇中心幼儿园建设，确保育才镇中心幼儿园建成招生，落实社会办幼儿园教育财政补贴。新增普通高中学位270个，确保市四中新校区按期招生，通过联合办学等方式引进和创建优质学校。进一步提高中职学校办学水平，推进琼州学院三亚校区等高校建设。全面实施农村和城乡结合部中小学生营养工程，落实“两免一补”、“三免一补”等惠民政策。强化学校及校车安全管理。

（二）进一步加强就业工作。加强公共就业服务体系建设，多渠道促进高校毕业生就业，解决好下岗失业人员、被征地农民、拆迁居民等人员的就业。今年要新增城镇就业岗位2.6万个，新增农村劳动力转移就业1万人。积极开展创业培训，加强创业服务，继续完善小额担保贷款制度，加大对小微型企业的资金支持力度。规范企业招用工行为，加强劳动合同管理和劳动保障监察。

（三）千方百计增加居民收入。健全企业工资支付保障机制，落实最低工资标准。进一步改进征地补偿机制，通过配建出租屋、商业房等方式增加群众财产性收入。鼓励开发休闲农业、乡村旅游项目，拓宽农民增收渠道。加快实施以“整村推进”为重点的贫困乡村基础设施建设，开展连片开发和扶贫互助资金试点工作，扩大产业化扶贫覆盖面，在年内实现2900名贫困人口脱贫。

（四）努力保持物价总水平基本稳定。加强“菜篮子”工程建设，提高主要农副产品产量。开工建设吉阳农产品综合批发市场，加快农贸市场升级改造，加强农超对接工作，开展鲜活农产品直供直销配送体系建设试点，完善农副产品平价商店和平价专营区运营管理。进一步强化价格监测预警和市场价格监管，适时启动物价上涨联动补贴。抓好重要生活必需品储备工作。

（五）不断完善社会保障体系。加快构建覆盖城乡的社保体系，城镇职工五项保险参保率要保持在98%以上，城镇居民养老保险、新型农村养老保险参保率要保持在95%以上，失地农民养老保险要做到应保尽保，城乡居民医疗保险参保率要保持99%。继续完善城乡低保标准动态增长机制。积极开展社会救济和社会福利工作，开工建设三亚残疾儿童康复中心，大力推进敬老院项目建设。

（六）强化住房保障工作。今年全市要确保建成各类保障性住房6444套，要新开工建设5300套，并完成农村危房改造1500户。要继续抓好保障性住房审核分配，加强资格审查，强化入住后的动态管理。

（七）提升医疗卫生服务水平。加快推进市人民医院整体改扩建二期、市中医院三期工程，开工实施市妇幼保健院整体搬迁建设项目。支持重点医院和基层卫生服务机构完善医疗设备。发挥解放军总医院海南分院的帮带作用，加强公立医院对基层医疗机构的对口支援。以破除“以药补医”机制为关键环节，进一步深化公立医院改革。强化药品和医疗器械安全监管。提高城乡基层公共卫生服务经费保障标准，积极开展疾病预防和保健工作。

（八）加强和创新社会管理。积极推进社区便民服务站建设，扩大社区管理“网格负责制”覆盖面，强化社区自治与服务功能。进一步完善社会治安防控体系，推进警务信息化建设。严格落实安全生产责任制，加强事故多发行业和领域专项整治。继续推进餐饮服务食品安全示范工程建设，切实提高食品安全监管水平。严格落实信访工作责任制，加强信访事项督查督办，定期梳理并主动回应群体性利益诉求，构建基层矛盾调解综合服务平台。加强国防教育、国防动员和国防后备力量建设，广泛开展双拥共建活动。加快发展妇女儿童、青少年、老龄、残疾人事业，继续做好民族宗教、外事侨务、人口计生、统计、档案、口岸等工作。

五、继续推进服务型政府建设

进一步转变政府职能，加强制度建设和管理创新，落实改进工作作风的各项规定，不断提升行政效能，增强服务能力。

（一）坚持依法行政。严格按照法定权限和程序履职用权，自觉接受人大法律监督、工作监督和政协民主监督。坚决执行市人大及其常委会的决议、决定，认真听取各民主党派、各人民团体和各界人士的意见、建议。完善重大决策听证、重要事项公示等制度，注重公众参与，保障人民群众的知情权和参与权。进一步完善行政执法体制，规范行政执法程序。

（二）着力提升行政效能。深化行政审批制度改革，落实“三集中”审批机制，依托市政府政务服务中心，规范流程，简化环节，提高效率，限时办结。积极开展重点项目并联审批，推行网上预审机制。继续完善土地两级储备、收益分享等配套政策，加快人、财、事权下放，逐步理顺区镇管理体制。积极创造条件，推动海棠湾撤镇设区等区划调整相关工作。继续分类推进事业单位改革。加强政府工作督查和绩效考评，落实问责制度。办好“政风行风热线”，建立健全行政投诉工作网络。

（三）加强政府廉政建设。深入推进廉政风险防控管理，继续加强对重要岗位、重点领域行政权力的监督和制约。进一步完善行政审批电子监察系统建设。积极推动工程建设、政府采购、土地出让、产权物权交易等四大类公共资源交易统一管理。坚持依法审计、独立审计，加强审计整改和责任追究。加大政务公开力度，特别是抓好涉及民生的重点资金、重点项目公开，扩大民生与公共服务类信息公开，抓好行政审批、专项资金管理使用等5个热点领域的动态公开，接受群众监督。严肃查处各类违法违纪案件，坚决纠正损害群众利益的不正之风。

各位代表，新的一年，三亚面临着新的任务和挑战。让我们紧密团结在以习近平同志为核心的党中央周围，深入贯彻落实党的十八大精神，在中共三亚市委的领导下，开拓创新，真抓实干，为建设更加卓越、更加和谐的三亚而努力奋斗！
